# Transient Receptor Potential Ankyrin 1 (TRPA1) Mediated LPS-Induced Inflammation in Periodontal Ligament Stem Cells by Inhibiting the Phosphorylation of JNK

**DOI:** 10.1155/sci/7461604

**Published:** 2024-12-20

**Authors:** Xian Wang, Xin Chen, Jie Gao, Zuolin Jin

**Affiliations:** State Key Laboratory of Oral and Maxillofacial Reconstruction and Regeneration, National Clinical Research Center for Oral Diseases, Shaanxi Clinical Research Center for Oral Diseases, Department of Orthodontics, School of Stomatology, The Fourth Military Medical University, No. 169 Changle West Road, Xi'an 710032, China

**Keywords:** JNK, PDLSCs, TRPA1

## Abstract

Transient receptor potential ankyrin 1 (TRPA1) molecule is an important type of transient receptor potential (TRP) cation channels, which can cause extracellular Ca^2+^ to flow into cells after activation. TRPA1 plays an important role in acute and chronic pain, inflammation, kidney disease, cough and asthma, osteoarthritis, cardiovascular disease, obesity, diabetes, and other diseases. In this study, the expression of interleukin (IL)-1*β*, IL-6, and IL-8 in periodontal ligament stem cells (PDLSCs) treated by lipopolysaccharide (LPS) and the effect of LPS on PDLSCS proliferation were detected. Meanwhile, the change in TRPA1 expression in PDLSCs treated by LPS was also assessed. By knocking down the expression of TRPA1 and using the TRPA1 antagonist HC-030031, the expression of IL-1*β*, IL-6, and IL-8 in PDLSCs treated by LPS was downregulated. After LPS stimulation, the proliferation ability of PDLSCs decreased, the gene expression and secretion of IL-1*β*, IL-6, and IL-8 increased and the gene and protein expression of TRPA1 were upregulated. Reducing the expression of TRPA1 can effectively inhibit the increase of gene expression of IL-1*β*, IL-6, and IL-8 after LPS stimulation, and pretreatment of PDLSCs with HC-030031 can also achieve the above effect. And research has found that HC-030031 can inhibit the phosphorylation level of JNK in PDLSCs treated by LPS. The use of JNK inhibitor JNK-IN-8 can also reduce the expression of IL-1*β*, IL-6, and IL-8 in PDLSCs. Finally, this study found LPS could cause the upregulation of TRPA1, and the inhibition of TRPA1 could produce an anti-inflammatory effect in PDLSCs treated by LPS due to its inhibition of JNK phosphorylation.

## 1. Introduction

Periodontitis is a disease characterized by the destruction of alveolar bone and the loss of periodontal support tissue, affecting over 3 billion people worldwide [1]. The periodontal ligament (PDL) plays a crucial role in the development of periodontitis [2]. In a healthy state, the homeostasis of the PDL can maintain normal metabolism of periodontal tissue and repair damage [3]. As an important source of cell differentiation and proliferation in the PDL, periodontal ligament stem cells (PDLSCs) play a crucial role in the normal physiological function of the PDL [4]. However, for patients with periodontitis, under the long-term inflammatory microenvironment, the function of the PDL is affected and destroyed, resulting in a significant loss of periodontal support tissue [5]. The inflammatory microenvironment can stimulate the secretion of proinflammatory cytokines (such as interleukin (IL)-1*β*, IL-6, IL-8, etc.) [6–8], the production of these proinflammatory cytokines can cause further periodontal tissue damage, and studies have shown that PDLSCs derived from inflammatory tissues have increased proliferation ability compared to PDLSCs derived from healthy tissues, but decreased osteogenic differentiation ability [9]. Therefore, how to restore the biological function of PDLSCs under long-term inflammatory stimulation has become a hot topic in the treatment of periodontal disease.

In previous studies, our research team found that the gene expression of transient receptor potential ankyrin 1 (TRPA1) was significantly enhanced in PDLSCs derived from inflammatory tissues [10], but the specific reasons and related effects are still poorly understood. TRPA1 molecule is an important type of TRP cation channels [11], which can cause extracellular Ca^2+^ to flow into cells after activation [12]. TRPA1 plays an important role in acute and chronic pain [13], inflammation [14], kidney disease [15], cough and asthma [16–18], osteoarthritis [19], cardiovascular disease [20], obesity [21, 22], diabetes [21], and other diseases. The application of TRPA1 antagonist in these diseases can achieve good therapeutic effects. For example, HC-030031 can treat allergic rhinitis and also help regulate the anxiety behavior of depressed mice. GDC-0334 can inhibit allergic-induced pulmonary neurogenic inflammation [23]. GRC-17536 has been used to treat respiratory diseases and diabetes peripheral neuropathy [24, 25]. The main function of TRPA1 antagonists is to block calcium flow, thereby preventing a series of physiological changes within cells, such as reactive oxygen species (ROS) generation [26], endoplasmic reticulum (ER) and mitochondrial stress [27], and the generation of proinflammatory cytokines [28]. Therefore, TRPA1 has been widely recognized as a significant target for the treatment of related diseases.

TRPA1 is expressed in various odontogenic cells, such as PDLCs [27], dental pulp cells [29], dental pulp fibroblasts [30], and odontoblasts [31]. Its main function is to act as a mechanical force-sensitive channel [12] and affect inflammatory pain [32]. Reduced expression of TRPA1 in PDLSCs can alleviate oxidative stress and apoptosis through PERK/elF2*α*/ATF-4/CHOP pathway [27]. While in PDL, TRPA1 acts as a thermosensitive channel to affect the production of IL-6 and IL-8 [33]. TRPA1 in human odontoblast-like cells [34] is involved in lipopolysaccharide (LPS)-induced immune responses. Other molecules in the TRP family, such as TRPV1, TRPV4, and TRPM3, can regulate alveolar bone remodeling by regulating the RANKL/OPG signaling pathway, and mechanical forces can regulate the osteogenic differentiation of PDLSCs through TRPV4 [35–37]. However, the expression of TRPA1 and its related effects in PDLSCs affected by the inflammatory microenvironment are still known little.

Therefore, in this study, the expression changes of TRPA1 were detected at the gene and protein levels by simulating the inflammatory microenvironment using LPS. We explored the impact of TRPA1 on the expression of proinflammatory cytokines and related molecular mechanisms in the inflammatory microenvironment, thus providing a new method for the subsequent treatment of periodontitis.

## 2. Materials and Methods

### 2.1. Isolation and Culture of Primary Human PDLSCs

PDLSCs were cultured from four patients aged 31–37 years, who require extraction of premolars for orthodontic treatment. Ethical approval was obtained from the Medical Ethics Committee of Shaanxi Province (licence number: IRB-Rev-2020005). All patients were informed of the research project, and related informed consent forms were signed. Following the extraction, teeth were put in the *α*-minimum Eagle's medium (*α*-MEM) (HyClone, Logan, Utah, USA) contained 10% penicillin–streptomycin solution. The PDL was scraped off from the middle third of the root surface and then washed in Hank's balanced salt solution (HBSS). Collagenase I (1 mg/mL, Diyibio, Shanghai, China) was used to digest the PDL for 30 min. After digestion, the single-cell suspension was filtered through a 70 μm strainer and then maintained in *α*-MEM with 10% fetal bovine serum (FBS) (Gibco, Waltham, MA, USA), 100 U/mL penicillin–streptomycin solution (Beyotime, Shanghai, China) at 37°C in 5% CO_2_. The medium was changed per 3 days. After reaching the 80%–90% of confluence, the cells were detached with 0.05% trypsin/EDTA (NCM Biotech, Suzhou, China) and passaged. All experiments were performed using PDLSCs between passage four and six to avoid changes in cell behavior caused by prolonged culture.

### 2.2. In Vitro Treatment of Primary Human PDLSCs

PDLSCs were seeded in six-well plate with 2 mL medium. The experimental wells were treated with 2 μL LPS in the dose of 1 μg/mL for 3 h or 12 h and incubated at 37°C in 5% CO_2_. In addition, PDLSCs were also treated with LPS following pretreated with the specific TRPA1 antagonist HC-030031 (MedChem Express, NJ, USA), JNK inhibitor JNK-IN-8 (MedChem Express, NJ, USA), or TRPA1 siRNA (GenePharma, Shanghai, China) to detect the change of inflammatory factors in PDLSCs and culture supernatant.

### 2.3. Transfection of siRNA

To inhibit the TRPA1 expression in PDLSCs, the TRPA1 and vehicle control siRNA were used to transfect. GP transfect mate (GenePharma, Shanghai, China) were added in medium according the manufacturer's instruction which was conducive to transfection. siRNA was diluted in culture medium then mixed with transfection reagent. The cells were incubated at 37°C in 5% CO_2_ with siRNA for 6 h.

### 2.4. Flow Cytometry

The PDLSCs were detached with 0.05% trypsin/EDTA, then harvested and washed with cold phosphate buffer solution (PBS) 3 times. After the last time washing, the cells were suspended by PBS. Then, cells were labeled (60 min in the dark at room temperature (RT)) with the following monoclonal antibodies conjugated with fluorescent dyes: CD73 (BioLegend, 344017), CD90 (BioLegend, 328106), CD105 (BioLegend, 323214), CD11b (BioLegend, 379902), CD19 (BioLegend, 302202), CD45 (BioLegend, 361902), STRO-1 (Abcam, ab190282), and HLA-DR (BioLegend, 327002).

### 2.5. Colony-Forming Assays

PDLSCs (1000 per dish) were seeded and incubated with LPS (1 μg/mL) in 10 cm dishes for 2 weeks. The samples were fixed with 4% paraformaldehyde solution (Coolaber, SL1830, China) for 30 min and stained with crystal violet solution (0.1%, MedChem Express, NJ, USA). After washing 3 times with PBS, the number of colonies in each dish was counted under a microscope.

### 2.6. Cell Proliferation Assay

PDLSCs (800 per well) were seeded and incubated with LPS (1 μg/mL) in 96-wells plate for 8 days. Cell proliferation was monitored using a CCK-8 kit (NCM Biotech, Suzhou, China) according to the manufacturer's instructions. The cells was incubated with CCK-8 reagent at 37°C for 2 h, then measured the absorbance of 450 nm using a microplate reader (Biotek Epoch, USA).

### 2.7. Osteogenic and Adipogenic Differentiation

Cells were incubated with OriCell human bone marrow mesenchymal stem cell osteogenic differentiation medium (HUXMX-90021) for 21 days, and the solution was changed accoding to the manufacturer's instructions. Cells were fixed with 4% paraformaldehyde and stained with alizarin red. Similarly, cells were incubated with OriCell human bone marrow mesenchymal stem cell adipogenic differentiation medium (HUXMX-90031) for 14 days according to the manufacturer's instructions. Fixed with 4% paraformaldehyde and stained with oil red O solution. Observation of staining results using a body microscope and an inverted microscope. These experiments were repeated three times.

### 2.8. Real-Time PCR

Total RNA was extracted using Trizol reagent and SteadPure RNA extraction kit (Accurate Biotechnology, Hunan, China) and cDNA was synthesized from 2.5 μg of total RNA using Evo M-MLV RT Premix for quantitative polymerase chain reaction (qPCR) (Accurate Biotechnology, Hunan, China). Primers were designed by Accurate Biotechnology (Hunan, China) (Table 1). RNA expression was measured with SYBR Green Premix Pro Taq HS qPCR kit (Accurate Biotechnology, Hunan, China) using 7500 real-time PCR detection system (Applied Biosystems, CA, USA). The data were analyzed using the 2^−ΔΔ*Ct*^ relative expression method. All experiments were repeated three times.

### 2.9. Western Blotting

Cells were lysed with RIPA (Proteintech, Wuhan, China) mixed with protease and phosphatase inhibitor cocktail (MedChemExpress, NJ, USA). The total protein levels were quantified with a bicinchoninic acid (BCA) protein assay kit (AccuRef Scientific, Xi'an, China). The protein was separated by 10% sodium dodecyl sulfate (SDS)–polyacrylamide gel (AccuRef Scientific, Xi'an, China) with 20 μg of protein sample in each well. Then the protein was transferred onto polyvinylidene difluoride (PVDF) membranes (Millipore, MA, USA). After being blocked in rapid blocking buffer (AccuRef Scientific, Xi'an, China) for 10 min, the membranes were washed in tris-borate-sodium Tween-20 (TBST) buffer (AccuRef Scientific, Xi'an, China) for 10 min and incubated overnight at 4°C with primary antibodies as following: anti-TRPA1 (NB110-4076; Novus), anti-ERK1/2 (11257-1-AP; Proteintech), anti-phospho-ERK1/2 (28733-1-AP; Proteintech), anti-JNK (66210-1-lg; Proteintech), anti-phospho-JNK (80024-1-RR; Proteintech), and anti-glyceraldehyde-3-phosphate dehydrogenase (GAPDH) (3683S, Cell Signaling Technology). Then, the membranes were incubated with an horseradish peroxidase (HRP)-conjugated goat antimouse or goat antirabbit IgG (1:10000; SA00001-1, SA00001-2, Proteintech), and the protein bands were detected by super ECL detection reagent kit (AccuRef Scientific, Xi'an, China). The relative density of results was measured by ImageJ software. Each experiment was repeated 3 times.

### 2.10. Quantification of Inflammatory Factors

IL-1*β*, IL-6, and IL-8 levels in the culture medium were determined using human IL-1*β*, IL-6, and IL-8 enzyme-linked immunosorbent assay (Elisa) kits (Proteintech, Wuhan, China), all steps were followed by the manufacturer's instructions.

### 2.11. Statistical Analysis

GraphPad Prism 8.0.2 was used for statistical analysis and graphic production. Data are expressed as the mean ± standard deviation (SD). Student's *t*-test was used for data analysis between two samples, and one-way analysis of variance (ANOVA) was used for multiple samples. Significance levels were determined by (*⁣*^*∗*^)*p*  < 0.05, (*⁣*^*∗∗*^)*p*  < 0.01, (*⁣*^*∗∗∗*^)*p*  < 0.001, and (*⁣*^*∗∗∗∗*^) *p*  < 0.0001.

## 3. Results and Discussion

### 3.1. Isolation, Culture, Identification, and Characterization of PDLSCs

**Figure 1:**
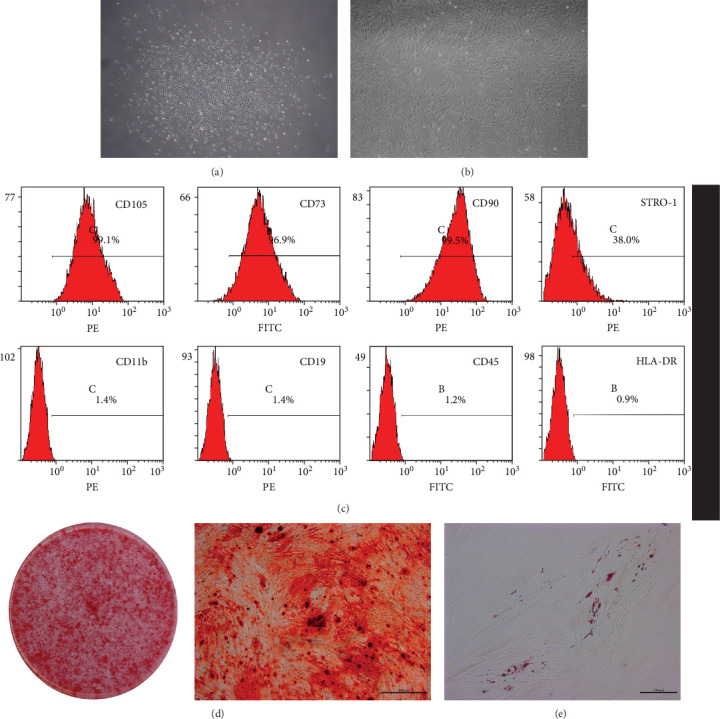
Isolation, cultivation, and identification of PDLSCs. P0 (A) and P3 (B) showed a typical fibroblast-like spindle appearance. Flow cytometry indicated that PDLSCs express markers of MSCs, CD73, CD90, CD105, and do not express CD11b, CD19, CD45, and HLA-DR (C). Alizarin red staining of PDLSCs under osteogenic induction medium for 21 (D). Oil red O staining of PDLSCs under adipogenic induction medium for 14 (E). B in panel (C) indicates the FITC channel of flow cytometry and C in panel (C) indicates the PE channel of flow cytometry. PDLSC, periodontal ligament stem cell.

PDLSCs were isolated successfully and cultured in a six-well plate. When the primary cells formed cell colonies (Figure 1A), the cells were passaged into 10 cm culture dish. PDLSCs were spindle-shaped like fibroblasts (Figure 1B). Flow cytometry indicated that PDLSCs exhibited stem cell characteristics consistent with MSCs (Figure 1C). After 3 weeks osteogenic induction and 2 weeks adipogenic induction, the mineralized nodules and fat droplets were observed by Alizarin red and oil red O staining (Figure 1D, E), so that the multipotentiality of isolated PDLSCs were confirmed and the PDLSCs could be used in following experiments.

### 3.2. LPS Inhibits the Proliferation of PDLSCs and Promotes the Expression of Pro-Inflammatory Cytokine IL-1*β*, IL-6, and IL-8

**Figure 2:**
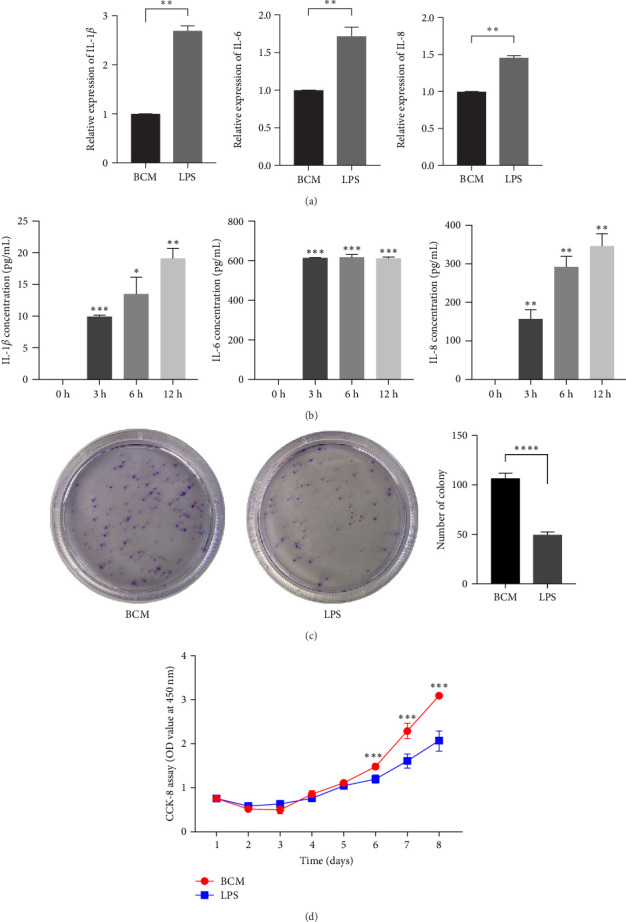
LPS promotes the expression of proinflammatory cytokine IL-1*β*, IL-6, IL-8 and inhibits the proliferation of PDLSCs. Human PDLSCs were stimulated with a LPS (1 μg/mL) for 12 h, IL-1*β*, IL-6, and IL-8 mRNA was measured by real-time PCR (A). In addition, culture media of PDLSCs stimulated by LPS at different times were collected, IL-1*β*, IL-6, and IL-8 concentrations in the culture media were measured by immunoassay and the results are expressed as mean ± SEM (B). Image of crystal violet staining for the colony-forming unit assay after cultivation 7 days (C). Image of cell proliferation assay of PDLSCs treated by LPS for 8days (D). *⁣*^*∗*^*p*  < 0.05, *⁣*^*∗∗*^*p*  < 0.01, *⁣*^*∗∗∗*^*p*  < 0.001,*⁣*^*∗∗∗∗*^*p*  < 0.0001. IL, interleukin; LPS, lipopolysaccharide; PDLSC, periodontal ligament stem cell.

LPS (1 μg/mL) was added into the culture medium to evaluate whether LPS affected the expression of proinflammatory cytokines in PDLSCs. 6 h after cultivation in the culture medium with LPS, the results showed that LPS increased the expression of genes of proinflammatory cytokines (IL-1*β*, IL-6, and IL-8) (Figure 2A) and the concentrations of IL-1*β* and IL-8 in medium increased as time prolonged, but IL-6 showed high concentration from the beginning (Figure 2B). To confirm that LPS can affect the proliferation of PDLSCs, cell proliferation assay and colony-forming assays were conducted in the study. The results revealed that cell proliferation of PDLSCs was reduced by LPS (1 μg/mL) (Figure 2C, D).

### 3.3. The Expression of IL-1*β*, IL-6, and IL-8 Were Downregulated by Gene Silencing of TRPA1

**Figure 3:**
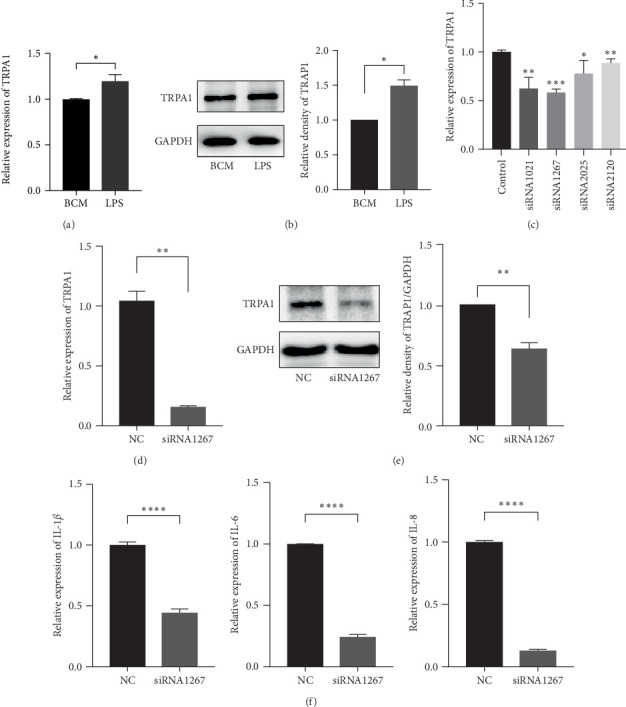
The expression of IL-1*β*, IL-6, and IL-8 were downregulated by gene silencing of TRPA1. PDLSCs were stimulated with LPS (1 μg/mL) for 12 h, the mRNA (A) and protein (B) expression of TRPA1 was measured by real-time PCR and western blot. Four siRNAs targeting TRPA1 were designed to transfect PDLSCs to lower TRPA1. After 1 day of transfection, the gene-silencing effect was measured by real-time PCR (C). PDLSCs were transfected by siRNA 1267 for 1 day, the mRNA and protein expression of TRPA1 were measured by real-time PCR and western blot (D and E). SiRNA 1267 PDLSCs and siNC PDLSCs were cultured with LPS 12 h after 2 days of transfection, IL-1*β*, IL-6, and IL-8 mRNA were measured by real-time PCR (F). The results are expressed as mean ± SEM. *⁣*^*∗*^*p*  < 0.05, *⁣*^*∗∗*^*p*  < 0.01,*⁣*^*∗∗∗*^*p*  < 0.001,*⁣*^*∗∗∗∗*^*p*  < 0.0001. IL, interleukin; LPS, lipopolysaccharide; PDLSC, periodontal ligament stem cell.

To study the change of TRPA1 in LPS-mediated elevation of proinflammatory cytokines in PDLSCs, the expression of total TRPA1 in both mRNA and protein level was analyzed with or without LPS treatment. The results of real-time PCR and western blot indicated LPS increased the expression of TRPA1 in PDLSCs (Figure 3A, B). Then we knocked down endogenous TRPA1 expression in PDLSCs by siRNAs. Four siRNAs targeting TRPA1 were designed to transfect PDLSCs to lower TRPA1 (Supporting Information 1: Figure S1). The results showed that siRNA 1267 had the best gene-silencing effect (Figure 3C), so we chose siRNA 1267 to conduct the further experiments. Transfection of PDLSCs with siRNA1267, qPCR, and western blot confirmed that TRPA1 was effectively silenced (Figure 3D, E). siRNA 1267 PDLSCs and siNC PDLSCs were cultured with LPS 12 h after 2 days of transfection, as shown in Figure 3F, The silencing of TRPA1 in PDLSCs effectively suppressed the expression of IL-1*β*, IL-6, and IL-8.

### 3.4. Inhibition of TRPA1 Represses the Phosphorylation of JNK

**Figure 4:**
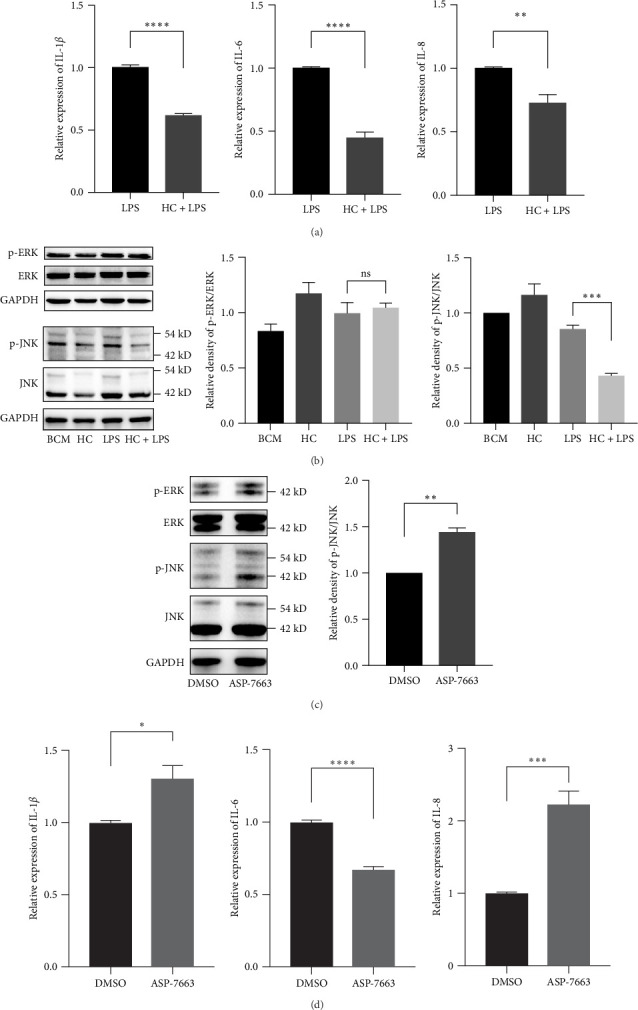
Inhibition of TRPA1 represses the phosphorylation of JNK. PDLSCs were pretreated by HC-030031 with or without for 3 h, followed by incubation by LPS (1 μg/mL) for 12 h, the expression of IL-1*β*, IL-6, and IL-8 were measured by real-time PCR (A). Then the expression of p-ERK, ERK, p-JNK, and JNK in PDLSCs treated by LPS (1 μg/mL) for 3 h were measured by western blot (B). TRPA1 agonist ASP-7663 was used to stimulate PDLSCs for 3 h, the protein levels of p-ERK, ERK, p-JNK, and JNK were measured by western blot and the gene expressions of IL-1*β*, IL-6, and IL-8 were measured by real-time PCR (C and D). The results are expressed as mean ± SEM. *⁣*^*∗*^*p*  < 0.05, *⁣*^*∗∗*^*p*  < 0.01, *⁣*^*∗∗∗*^*p*  < 0.001, *⁣*^*∗∗∗∗*^*p*  < 0.0001. IL, interleukin; LPS, lipopolysaccharide; PDLSC, periodontal ligament stem cell.

TRPA1 antagonist HC-030031 can effectively reduce the gene expression of IL-1*β*, IL-6, and IL-8 in PDLSCs under LPS stimulation for 12 h (Figure 4A). Mitogen-activated protein kinases (MAPKs) are classic inflammation-related signal, so we investigated phosphorylation of ERK and JNK under LPS stimulation and TRPA1 antagonist HC-030031. The results showed HC-030031 can effectively reduce phosphorylation of JNK in PDLSCs after LPS stimulation, but has no significant effect on ERK phosphorylation (Figure 4B). Then we used TRPA1 agonist ASP-7663 to investigate the change of JNK phosphorylation and gene expression of IL-1*β*, IL-6, and IL-8. The results showed that ASP-7663 can enhance the phosphorylation of JNK and promote the gene expression of IL-1*β*, IL-6, and IL-8 (Figure 4C, D), and HC-030031 can prevent the effect of LPS on the proliferation ability of PDLSCs (Supporting Informtion 2: Figure S2). The results suggested that phosphorylation of JNK played a role in the upregulation of these inflammatory cytokines.

### 3.5. Inhibition of the Phosphorylation of JNK Can Inhibit the Expression of Inflammatory Cytokines in PDLSCs Treated by LPS

**Figure 5:**
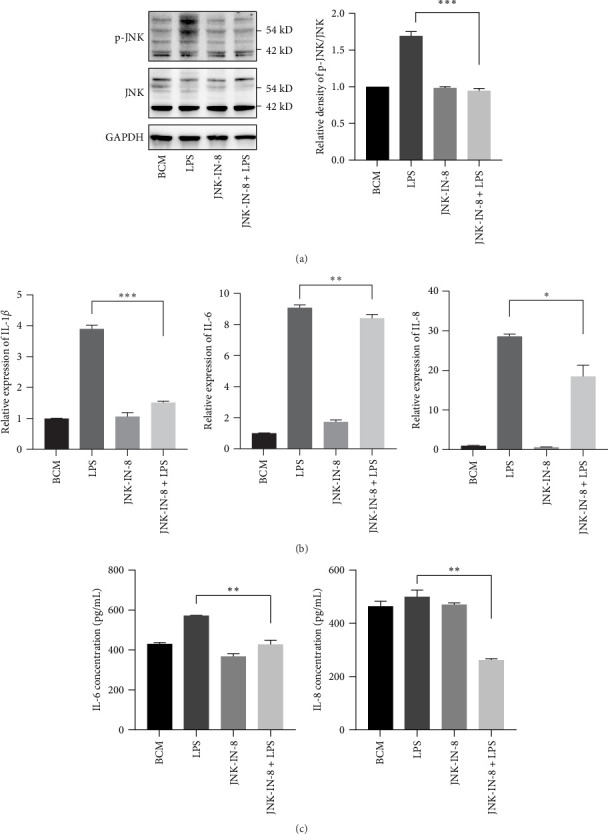
Inhibition of the phosphorylation of JNK can inhibit the expression of inflammatory cytokines in PDLSCs treated by LPS. PDLSCs were pretreated with or without JNK antagonist JNK-IN-8 for 3 h, followed by incubation by LPS (1 μg/mL) for 12 h, the protein levels of p-JNK and JNK were measured by western blot (A), the gene expressions of IL-1*β*, IL-6, and IL-8 were measured by real-time PCR and the concentrations of IL-6 and IL-8 in the culture media were measured by immunoassay (B and C). The results are expressed as mean ± SEM. *⁣*^*∗*^*p*  < 0.05, *⁣*^*∗∗*^*p*  < 0.01, *⁣*^*∗∗∗*^*p*  < 0.001, *⁣*^*∗∗∗∗*^*p*  < 0.0001. IL, interleukin; LPS, lipopolysaccharide; PDLSC, periodontal ligament stem cell.

To clarify the role of JNK in LPS-induced production of inflammatory cytokines in PDLSCs, we used JNK inhibitor JNK-IN-8 to block the phosphorylation of JNK. The results showed that JNK-IN-8 can effectively reduce the phosphorylation of JNK (Figure 5A) and the gene expression of IL-1*β*, IL-6, and IL-8(Figure 5B). The concentration of IL-6 and IL-8 in culture medium reduced (Figure 5C), but the concentration of IL-1*β* was too low and not detected. The results suggested that inhibiting JNK phosphorylation can effectively reduce the expression of inflammatory cytokines in PDLSCs stimulated by LPS.

## 4. Discussion

The results of the study found that changes in the expression of TRPA1 in PDLSCs can affect the expression of inflammatory cytokines in PDLSCs treated by LPS, and this regulatory effect is largely achieved by regulating JNK phosphorylation. In addition, the use of TRPA1 molecule antagonist HC-030031 can reduce the expression and secretion of inflammatory cytokines in PDLSCs treated by LPS.

PDLSCs are an important component of the metabolism and remodeling process of periodontal tissue. Under physiological conditions, there is a dynamic balance between bone resorption and remodeling in periodontal tissue, maintaining its homeostasis [3]. However, for patients with periodontitis, this homeostasis is disrupted, and the rate of alveolar bone absorption is significantly faster than the rate of alveolar bone remodeling, resulting in sustained loss of alveolar bone tissue. Patients with severe periodontitis often require orthodontic treatment after proper periodontal treatment to eliminate occlusal trauma and align teeth that have been displaced due to alveolar bone loss. However, in the process of orthodontic treatment, patients who have undergone periodontal treatment often require light force for tooth movement, and the ability of periodontal reconstruction is significantly weaker than that of healthy periodontal patients [9]. Further research has found that the biological functions of PDLSCs within the PDL have changed under long-term inflammatory microenvironment effects, and their differentiation ability has significantly weakened. To verify the effect of inflammatory microenvironment stimulation on PDLSCs, we added LPS as a stimulus in the culture medium to simulate the inflammatory microenvironment, and tested the proliferation ability and expression of inflammatory cytokines in the inflammatory microenvironment. In this study, we successfully showed that LPS stimulation can weaken the proliferation ability and increased the expression of IL-1*β*, IL-6, and IL-8 in PDLSCs.

In our previous research, we found that the expression of TRPA1 gene was significantly increased in PDLSCs derived form inflammatory tissue compared to PDLSCs derived from healthy tissue. TRPA1 is a member of the TRP family, first discovered in pulmonary fibroblasts in 1999 [38]. TRPA1 can perceive various physiological and chemical stimuli and participate in important life activities such as temperature regulation, hearing, taste perception, and so forth [39]. Research has shown that TRPA1 is an important regulatory factor in inflammatory diseases [40, 41], serving not only as a promoter of inflammatory response, but also as a director of inflammatory mediators [42].

In the inflammatory microenvironment, it can be directly or indirectly activated by inflammatory signaling molecules. There is already a lot of literature indicating that inflammatory factors can activate TRPA1. The expression of inflammatory cytokines such as IL-1*β*, IL-6, and IL-8 can be effectively treated by inhibiting TRPA1. In asthma models induced by tobacco extract and PM2.5, inhibiting TRPA1 can reduce the expression of IL-1*β* and IL-18 [43]. Inhibiting TRPA1 can inhibit the NLRP3/caspase-1 pathway during lung injury, thereby reducing IL-1*β* [44]. Activation of TRPA1 molecules in scalp skin keratinocytes can promote the release of IL-1 *α*, IL-1*β*, and PGE2 [45]; Inhibition of TRPA1 in Alzheimer's disease mice can be achieved through NF-*κ*B pathway reduces the expression of IL-1*β* and IL-6 in mice [28]; Inhibiting TRPA1 can reduce IL-6 and IL-1*β* and tumor necrosis factor (TNF)-*α* levels in patients with osteoarthritis [46]. LPS can activate pulmonary epithelial cells and increase calcium flow which causes an increase in IL-8 secretion through activating NADPH oxidase and MAPK/NF-*κ*B signaling pathway [47, 48]. In this study, after LPS stimulation of PDLSCs, the gene and protein expression of TRPA1 in PDLSCs increased and the gene expression and weakened the proliferation ability and increased the expression of IL-1*β*, IL-6, and IL-8. The application of siRNA to silence the expression of TRPA1 molecule in PDLSCs can effectively reduce the generation of inflammatory cytokines (IL-1*β*, IL-6, and IL-8) in PDLSCs under LPS stimulation; TRPA1 antagonist HC-030031 was used to pretreat PDLSCs 3 h in advance, followed by LPS stimulation of PDLSCs. The expression of IL-1*β*, IL-6, and IL-8 decreased significantly. This result indicates that LPS can stimulate and increase the expression of TRPA1 molecule in PDLSCs, and the activation of TRPA1 molecule can enhance the expression of IL-1*β*, IL-6, and IL-8 in PDLSCs.

At present, research suggests that the relationship between TRPA1 and inflammatory factors IL-1*β*, IL-6, and IL-8 is related to the inflammation-related signaling pathway TLR4/NF-*κ*B and JNK/MAPK [49–51], while there is also a mutual connection between NF-*κ*B and MAPK pathway. The activation of JNK molecules can directly affect NF-*κ*B phosphorylation and nuclear translocation [52]. Therefore, in this experiment, the expression of ERK, JNK molecules, and their phosphorylation levels in the MARP pathway were detected. It was found that under LPS stimulation, the phosphorylation expression of JNK molecules in PDLSCs was significantly increased, and HC-030031 could effectively block this process. To verify the effect of JNK molecule phosphorylation on the expression of inflammatory cytokines in PDLSCs under LPS stimulation, JNK inhibitor JNK-IN-8 was used to pretreat PDLSCs, the gene expression of proinflammatory cytokines (IL-1*β*, IL-6, and IL-8) and secretion of IL-6 and IL-8 were significantly reduced.

In this study, we demonstrated that LPS can stimulate high expression of TRPA1 molecule in PDLSCs, and enhance the expression of inflammatory cytokines IL-1*β*, IL-6, and IL-8 by activating TRPA1 molecule. Knocking down the expression of TRPA1 molecule using siRNA can effectively downregulate the production of inflammatory cytokines in PDLSCs stimulated by LPS. The inhibition of TRPA1 molecule antagonist HC-030031 can exert the effects of inflammatory inhibition due to its inhibition of JNK phosphorylation.

## 5. Conclusions

This study mainly investigated the role of TRPA1 in the process of LPS stimulation-induced PDLSCs, and preliminarily explored the relevant mechanisms. To provide a new strategic approach for restoring the biological characteristics of PDLSCs under the long-term inflammatory microenvironment.

## Figures and Tables

**Table 1:** Primers Used for qRT-PCR.

Gene	Primer	Sequence
*β*-actin	Forward	5′-TGGCACCCAGCACAATGAA−3′
	Reverse	5′-CTAAGTCATAGTCCGCCTAGAAGCA−3′

IL-1*β*	Forward	5′-GGTACATCAGCACCTCTCAAF−3′
	Reverse	5′-CACATTCAGCACAGGACTCTC−3′

IL-6	Forward	5′-GCCACTCACCTCTTCAGAACG−3′
	Reverse	5′-GTGCCTCTTTGCTGCTTTCA−3′

IL-8	Forward	5′-CACTGTGTGTAAACATGACTTCCAA−3′
	Reverse	5′-TGTGGTCCACTCTCAATCACTCTC−3′

TRPA1	Forward	5′-TTCAGTCTTCCCTCTCCTCAAAC−3′
	Reverse	5′-TCTCACATTCTTGGCACTCAGT−3′

## Data Availability

All the data generated or analyzed during this study are included in this article.

## Supporting Information

Supporting Information 1 Figure S1: The fluorescence plot of four siRNAs transfection efficiency.

Supporting Information 2 Figure S2: Image of cell proliferation assay of PDLSCs treated by HC-030031, LPS, and HC-030031 + LPS for 5 days. *⁣*^*∗*^*p* < 0.05, *⁣*^*∗∗*^*p* < 0.01, *⁣*^*∗∗∗*^*p* < 0.001.
